# Insomnia and circadian rhythm: a bibliometrics study and visualization analysis *via* CiteSpace

**DOI:** 10.3389/fneur.2023.1184302

**Published:** 2023-06-15

**Authors:** Qing-Yun He, Ning Dai, Meng Mao, Jie Ma, Qiao Wen, Dan-Dan Song, Yan Liu, Feng Li

**Affiliations:** ^1^Department of Diagnosis of Traditional Chinese Medicine, School of Traditional Chinese Medicine, Beijing University of Chinese Medicine, Beijing, China; ^2^Research Institutes, Xiyuan Hospital, China Academy of Chinese Medical Sciences, Beijing, China; ^3^Department of Ethnic Medicine, School of Traditional Chinese Medicine, Beijing University of Chinese Medicine, Beijing, China; ^4^Department of Brain Diseases, Third Affiliated Hospital, Beijing University of Chinese Medicine, Beijing, China; ^5^Scientific Research Center, School of Traditional Chinese Medicine, Beijing University of Chinese Medicine, Beijing, China

**Keywords:** bibliometrics, CiteSpace, visualization analysis, insomnia, circadian rhythm, bipolar disorder, light therapy, melatonin

## Abstract

**Objective:**

The present study aimed to use CiteSpace to analyze the status of insomnia and circadian rhythm, identify the hot spots and trends, and provide a basis for future study.

**Method:**

The Web of Science database was searched for studies related to insomnia and circadian from its inception to 14 April 2023. CiteSpace was used to generate online maps of collaboration between countries and authors and revealed hot spots and frontiers in insomnia and circadian rhythm.

**Results:**

We searched 4,696 publications related to insomnia and circadian rhythm. Bruno Etain was the most prolific author with most publications, i.e., with 24 articles. The USA and the University of California were the leading country and the top institution in this field of study, with 1,672 and 269 articles, respectively. There was active cooperation between institutions, countries, and authors. Hot topics focused on circadian rhythm sleep disorders, circadian clock, light therapy, melatonin, and bipolar disorder.

**Conclusion:**

Based on the CiteSpace results, we recommend a more active collaboration between various countries, institutions, and authors to conduct clinical and basic research related to insomnia and circadian rhythm. Ongoing research focuses on the interaction of insomnia with circadian rhythms and the corresponding pathways of clock genes and by extension, the role of circadian rhythms in disorders such as bipolar disorder. Modulation of circadian rhythms may be a hot spot for future insomnia therapies (such as light therapy and melatonin).

## 1. Background

Insomnia is a sleep disorder characterized by frequent and chronic difficulties in falling asleep or maintaining sleep, resulting in insufficient sleep satisfaction (1), with a prevalence of 50% of primary care patients (2). Insomnia not only decreases the duration and depth of sleep but also fails to eliminate fatigue, impairs daytime function, such as work efficiency, jeopardizes physical and mental health, and burdens social and economic development (3–5). The current treatment of insomnia is based on benzodiazepine agonist drugs such as eszopiclone, diazepam, and zopiclone. However, these drugs do not appear to be curative for recovering daytime function (6). In addition, long-term use of such drugs can lead to various adverse effects, such as anxiety, dizziness, reduced deep sleep, and daytime fatigue (7–9), all of which may be related to the circadian rhythm.

Circadian rhythm refers to the intrinsic rhythm of life activities in a 24-h cycle, which plays a vital role in sleep, the endocrine system, and the cardiovascular system (10). Sleep and circadian rhythm are equally significant in daily life (11–13). Sleep is regulated by a combination of circadian rhythm and sleep homeostasis. In 1982, Borbely proposed the “Two-Process Model” to explain the mechanism of sleep regulation, which suggests that sleep–wake is regulated by circadian rhythm and sleep homeostasis (14). The human need for sleep is not only regulated by the amount of previous sleep–wakefulness but also coregulated by circadian rhythms, which together determine the timing and tendency to sleep. In a sleep–wake cycle, with increasing waking time, sleep homeostasis prevails, which maintains sleep stability in the first half of the night; in the second half of the night, that is, the second half of sleep, the role of sleep homeostasis gradually decreases, and the regulation of circadian rhythms occupies a dominant position. Thus, in a sleep–wake cycle, the two complement each other and maintain sleep stability. Besides, circadian rhythm plays a significant role in daytime function (15). Often, poor daytime function is caused by the time of sleep in relation to the circadian phase and the resulting irregular sleep patterns. Therefore, the study of insomnia and circadian rhythms is vital for insomnia, which can expand treatment ideas, improve patients' daytime functions, and enhance their quality of life and work and study efficiency.

In 1969, Alan Pritchard, a famous British intelligence scientist, first introduced the term “Bibliometrics,” which marked the formal birth of bibliometrics. Bibliometrics is a multidisciplinary science that uses statistics to describe or illustrate the relationships between published studies. It analyzes published information (such as books, journal articles, datasets, and blogs) and their associated metadata (such as abstracts, keywords, and citations) to analyze all knowledge vehicles quantitatively. It is a comprehensive body of knowledge that integrates mathematics, statistics, and bibliography and focuses on quantification (16, 17). Through the use of bibliometric software, it generates interactive visual representations of complex structures for statistical analysis and interactive visual exploration. This comprehensive literature review of developments in a particular field not only gives researchers a foundation for analyzing disciplinary hot spots and development frontiers and predicting the direction of discipline development but also serves as a guide for hospital discipline construction and talent (18). CiteSpace, developed by Chaomei Chen, is a software that can be used for bibliometric research and visual analysis (19). It can visually reveal the dynamic development pattern of scientific knowledge and provide practical and valuable reference or guidance to assist researchers in actively tracking the development of academic fields by their own hands (20). CiteSpace contains three essential concepts: burst detection, betweenness centrality, and heterogeneous networks, which can label keywords, spot emerging patterns, and identify abrupt shifts in time. In this study, CiteSpace was used to analyze country of origin, author co-authorship, keyword co-occurrence, keyword cluster, keywords burst, and timeline in the field of insomnia and circadian rhythm to discuss the research hot spots and trends in insomnia and circadian rhythm and provide a basis for the study of the interaction regulation of the two topics.

## 2. Methods

### 2.1. Data sources and search strategies

**Figure 1:**
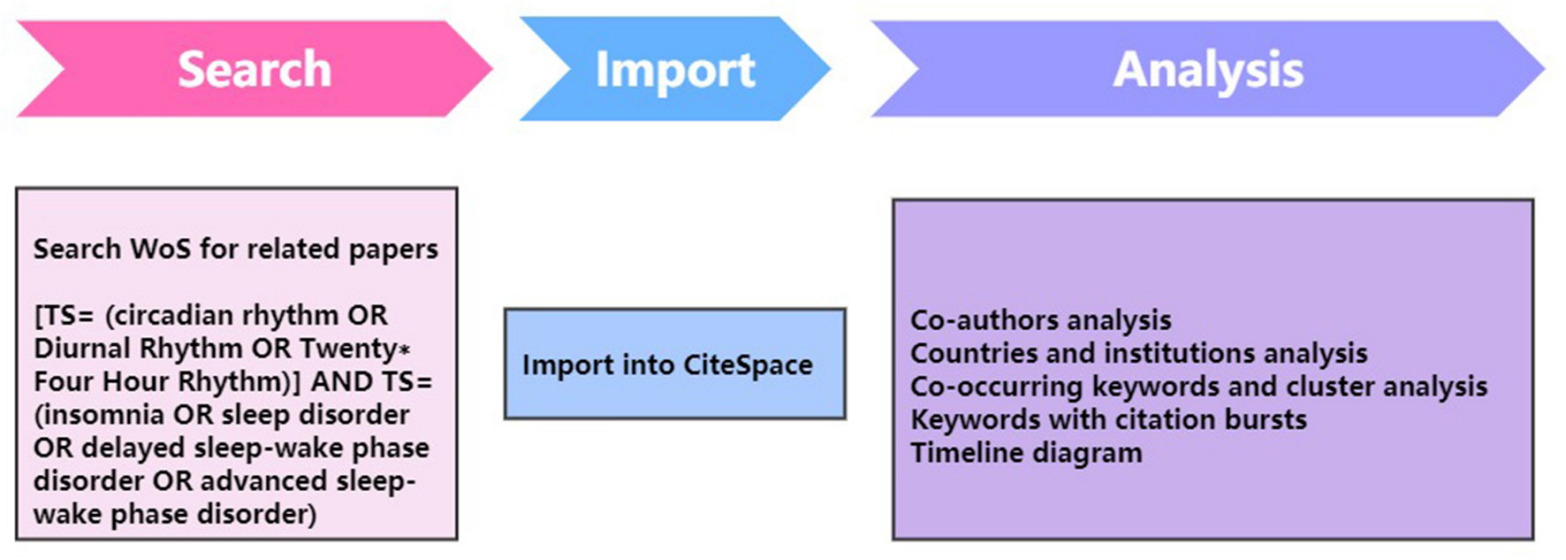
Flowchart for CiteSpace.

With more than 12,000 influential journals, the Web of Science (WoS) database is one of the most widely used academic databases. WoS is largely acknowledged as the most complete and trustworthy resource for bibliometric analysis compared to other databases such as Scopus and PubMed (21, 22). As shown in Figure 1, WoS was searched for studies in insomnia and circadian rhythm from the inception to 14 April 2023. The following terms were searched for in the topic: [TS= (circadian rhythm OR Diurnal Rhythm OR Twenty* Four Hour Rhythm)] AND TS= (insomnia OR sleep disorder OR delayed sleep-wake phase disorder OR advanced sleep-wake phase disorder). The language was restricted only to English, and only original articles and reviews were included.

### 2.2. Visualization analysis tool—CiteSpace

In this study, we used the 6.2.R2 version of CiteSpace software to analyze the current hot spots of insomnia and circadian rhythm research and to predict the research trends. In our study, we named the title of the literature obtained by searching WoS as “download_XXX.txt,” and after importing it into the CiteSpace software, we performed the analysis of keyword co-occurrence, keyword clustering, keyword burst, timeline, author co-authorship, country co-authorship, and institution co-authorship. The selection period for data processing ranged from 2000 to 2023, the time slice is 1 year, and the node selections are keyword, author, institution, and country with the rest being set to default settings (23).

## 3. Results

### 3.1. Bibliometric analysis of publication years

**Figure 2:**
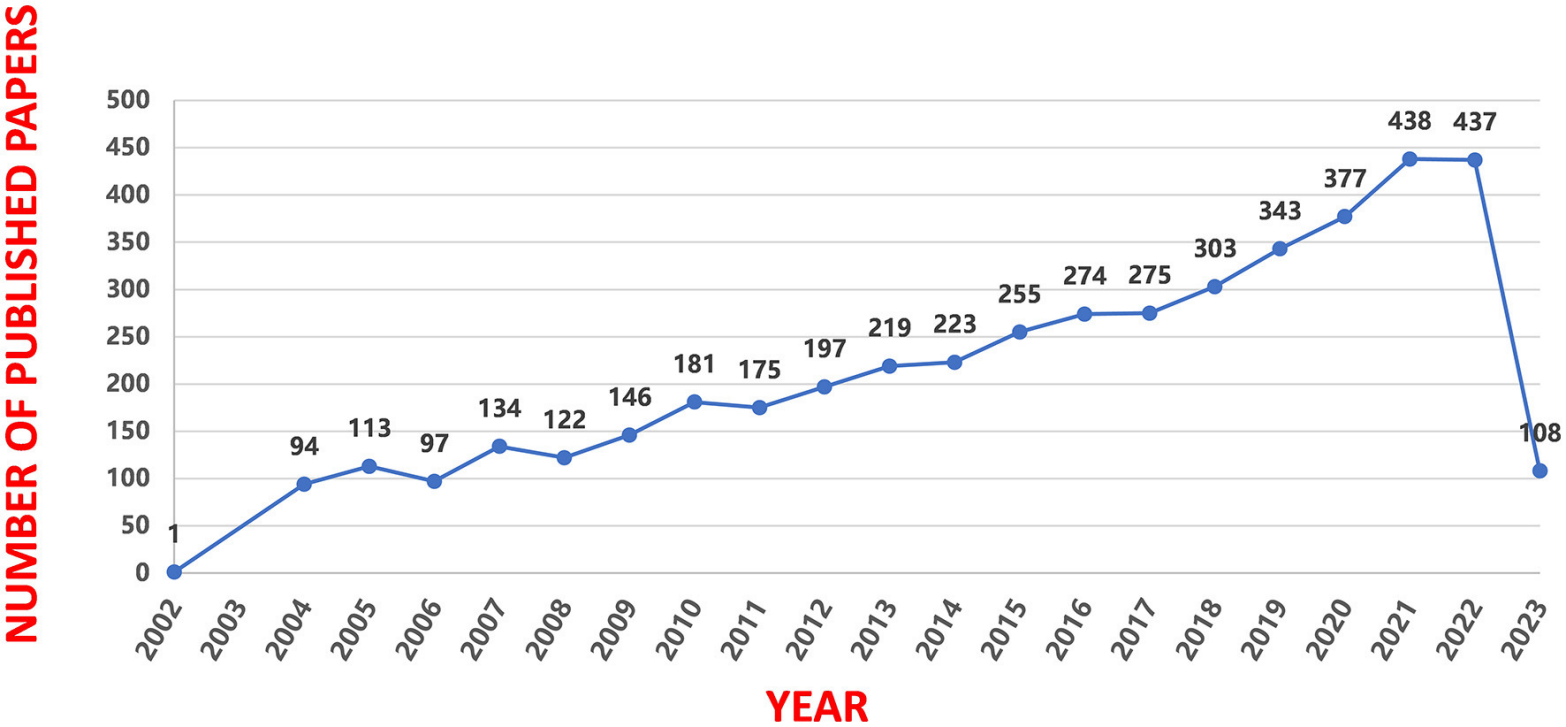
Annual trend chart of publications.

A total of 4,696 relevant articles were obtained after the search. As depicted in Figure 2, there have been an overall steady increase in articles on insomnia and circadian rhythm. The search was conducted from inception to April 2023, and there were relatively few articles available for this year during the time of study.

### 3.2. Bibliometric analysis of countries and institutions

**Figure 3:**
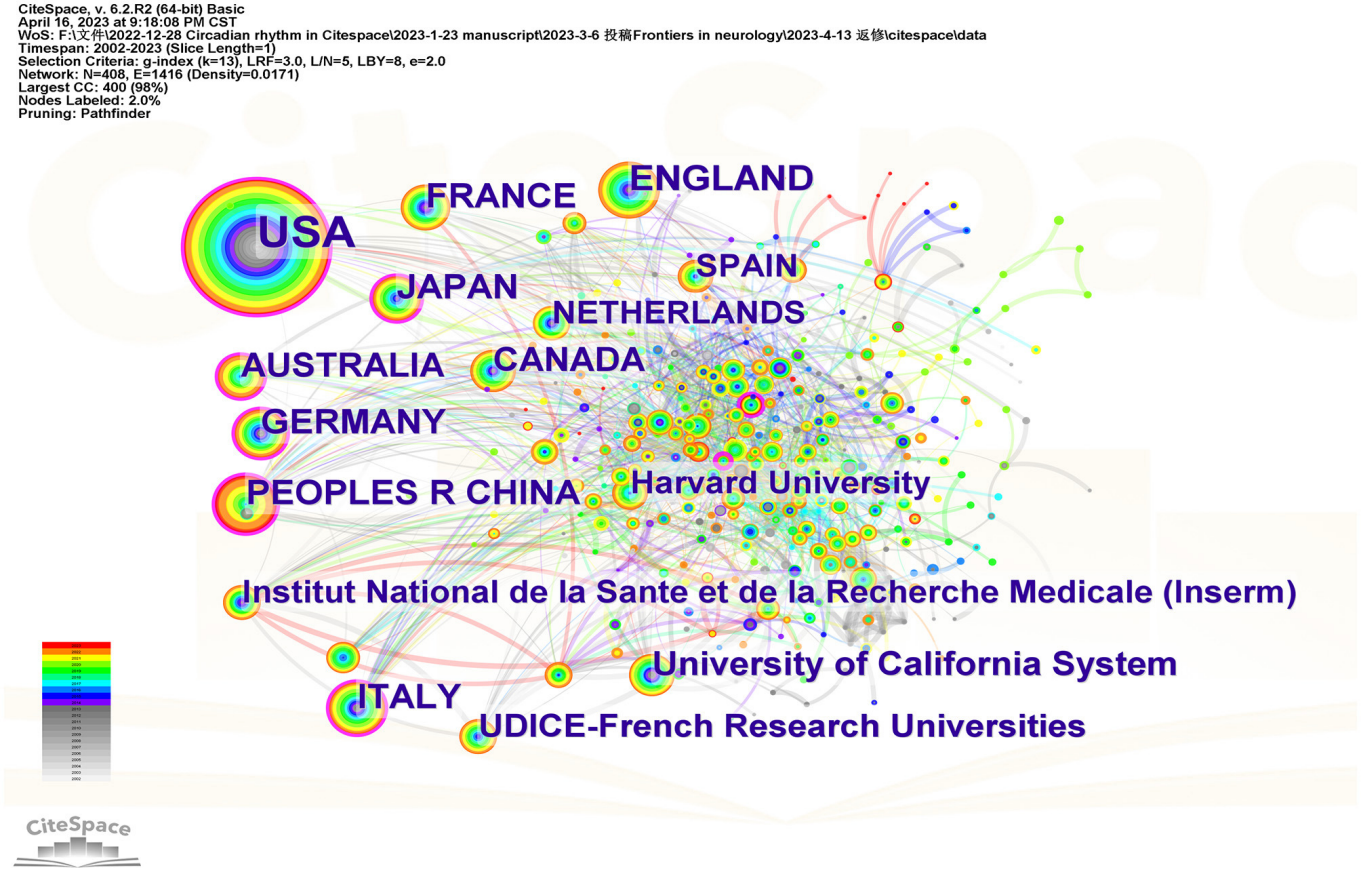
The network of countries and institutions in the study area of insomnia and circadian rhythm.

A total of 41 countries, regions, and institutions published articles concerning insomnia and circadian rhythm. As listed in Table 1, the top 10 countries and top 5 institutions contributed 4,405 (55.33%) and 1,033 (12.97%) studies, respectively. The top 10 countries were the USA, England, China, France, Italy, Germany, Japan, Canada, Australia, and the Netherlands. In addition, the University of California, UDICE-French Research Universities, Harvard University, Institut National de la Sante et de la Recherche Medicale (Inserm), and Harvard Medical School were among the top five institutions. The USA had the highest number of published studies, with 1,672 articles accounting for 21.00% of the total number of articles, which is much higher than the second-ranked country of England (396 articles), indicating that the USA had a dominant position in the field of insomnia and circadian rhythm. Furthermore, the University of California, which published the most articles, is also located in the USA. As shown in Figure 3, there was active cooperation between countries and institutions, particularly with the USA and European countries. As shown in the figure, the nodes of the USA, China, Australia, Italy, and Germany have a purple circle on the exterior, which means the centrality of these nodes was higher and these countries contributed more. Besides, they had closer cooperation with other countries in this field.

**Table 1:** The top 10 countries and top 5 institutions with most publications in the field of insomnia and circadian rhythm.

**Rank**	**Country**	***N* (%)**	**Institution**	***N* (%)**
1	USA	1,672 (21.00)	University of California	269 (3.38)
2	England	396 (4.97)	UDICE-French Research Universities	226 (2.84)
3	China	341 (4.28)	Harvard University	206 (2.59)
4	France	319 (4.01)	Institut National de la Sante et de la Recherche Medicale (Inserm)	180 (2.26)
5	Italy	318 (3.99)	Harvard Medical School	152 (2.59)
6	Germany	300 (3.77)		
7	Japan	291 (3.65)		
8	Canada	288 (3.62)		
9	Australia	275 (3.45)		
10	The Netherlands	205 (2.57)		

### 3.3. Bibliometric analysis of co-authors

**Figure 4:**
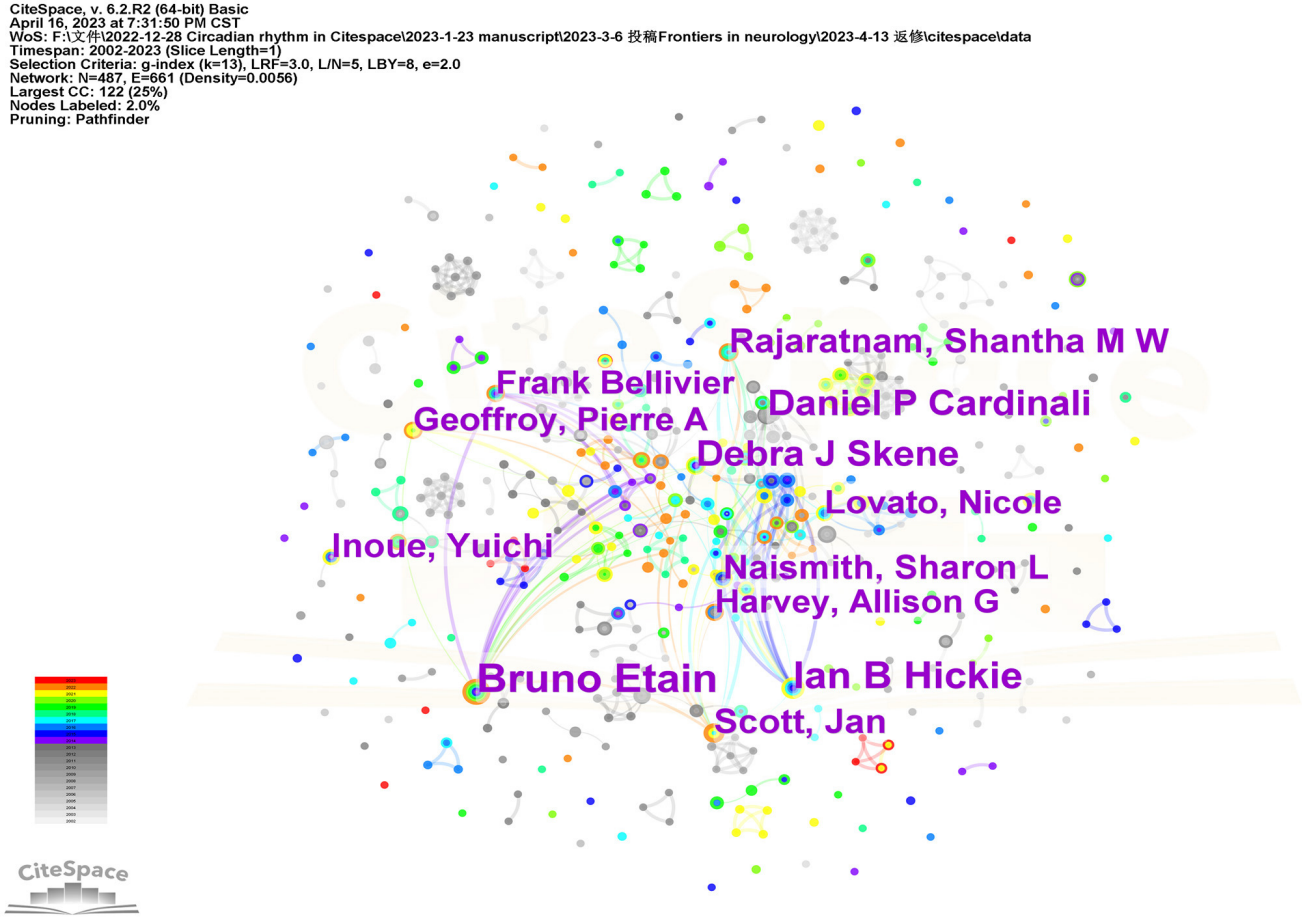
The network of co-authors in the study area of insomnia and circadian rhythm.

Figure 4 shows the network of co-author formed by CiteSpace. The top 10 authors contributed 153 articles (3.25%) (Table 2). The most prolific author was Bruno Etain from France; 24 of his articles were searched. His field of research focused on susceptibility factors to bipolar disorders, with a particular interest on sleep/circadian rhythm abnormalities, clinical and dimensional phenotypes, susceptibility genes, and childhood maltreatment. His article, *Genetic relationship between five psychiatric disorders estimated from genome-wide SNPs* (24), published in *Nature Genetics* in 2013 was cited 1,537 times as of 16 April 2023. The bolder line connecting distinct circles indicates greater collaboration among authors. As illustrated in Figure 4, the authors of this field typically shared consistent collaborative relationships with other authors.

**Table 2:** The top 10 authors of insomnia and circadian rhythm.

**Rank**	**Author**	**Count of articles**	**Year of the first article**
1	Bruno Etain	24	2011
2	Ian B Hickie	20	2014
3	Debra J Skene	16	2008
4	Daniel P Cardinali	16	2008
5	Scott, Jan	14	2017
6	Rajaratnam, Shantha M W	13	2010
7	Naismith, Sharon L	13	2013
8	Inoue, Yuichi	13	2008
9	Lovato, Nicole	12	2014
10	Harvey, Allison G	12	2011

### 3.4. Bibliometric analysis of co-occurring keywords and cluster

**Figure 5:**
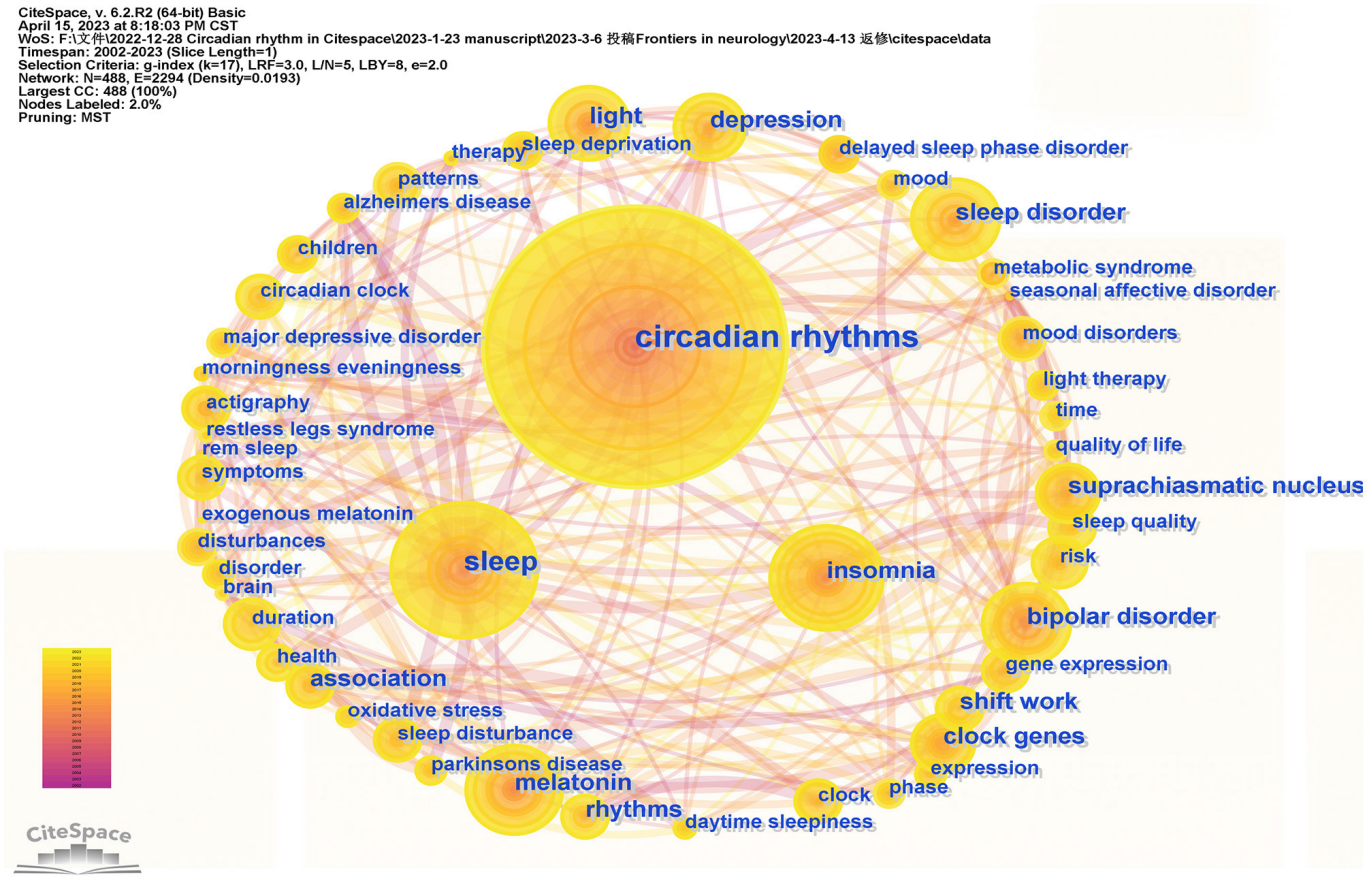
The network map of keywords.

Keyword co-occurrence can reflect the hot spots in the particular field. In the co-occurrence network map, the nodes indicate the corresponding keywords, the size of the nodes denotes the number of articles containing the keywords, and the connecting lines between the nodes indicate the relationship between the keywords. As shown in Figure 5, the connecting lines between each keyword are intricate, suggesting a complex connection between them. High-centrality keywords show the status and importance of the corresponding research content in the research field, while high-frequency keywords reflect a popular theme. As listed in Table 3, the top ten high-frequency keywords were as follows: “Circadian rhythms” (frequency: 2,374), “Sleep” (frequency: 779), “Insomnia” (frequency: 478), “Light” (frequency: 460), “Melatonin” (frequency: 456), “Sleep disorder” (frequency: 450), “Bipolar disorder” (frequency: 390), “Disorders” (frequency: 371), “Suprachiasmatic nucleus” (frequency: 310), and “Depression” (frequency: 283). The top 10 high centrality keywords were as follows: “Light” (centrality: 0.08), “Clock gene” (centrality: 0.06), “Sleep deprivation” (centrality: 0.06), “Secretion” (centrality: 0.06), “Double blind” (centrality: 0.05), “Alzheimers disease” (centrality: 0.05), “Seasonal affective disorder” (centrality: 0.05), “Actigraphy” (centrality: 0.05), “Delayed sleep phase disorder” (centrality: 0.05), and “Brain” (centrality: 0.05).

**Table 3:** Top 10 keywords in terms of frequency and centrality of insomnia and circadian rhythm.

**Rank**	**Frequency**	**Keywords**	**Centrality**	**Keywords**
1	2,374	Circadian rhythms	0.08	Light
2	779	Sleep	0.06	Clock gene
3	478	Insomnia	0.06	Sleep deprivation
4	460	Light	0.06	Secretion
5	456	Melatonin	0.05	Double blind
6	450	Sleep disorder	0.05	Alzheimers disease
7	390	Bipolar disorder	0.05	Seasonal affective disorder
8	371	Disorders	0.05	Actigraphy
9	310	Suprachiasmatic nucleus	0.05	Delayed sleep phase disorder
10	283	Depression	0.05	Brain

**Figure 6:**
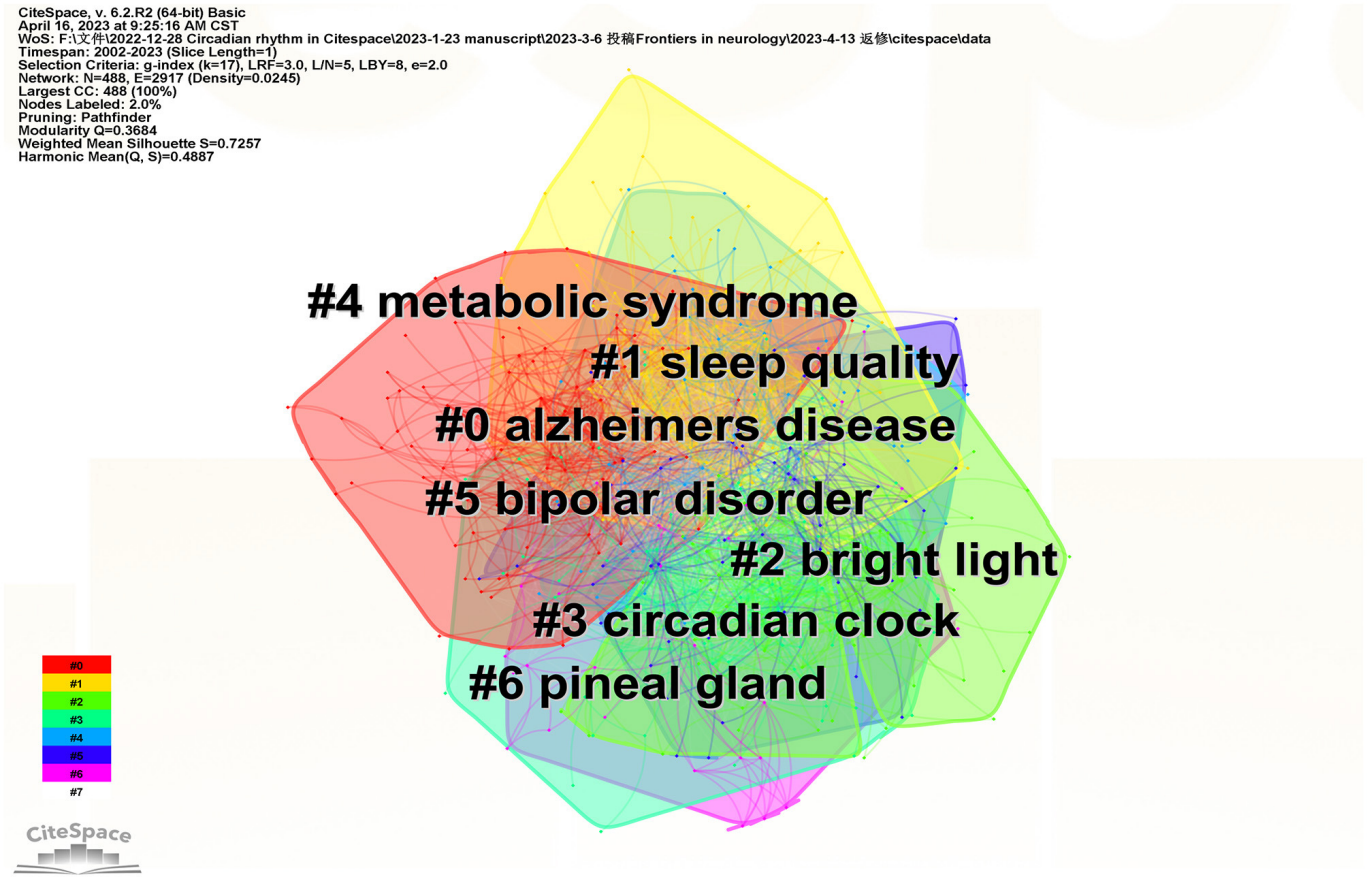
Keywords cluster analysis cooccurrence map.

Clustering analysis which could reveal the main topics was carried out for cooccurrence keywords *via* CiteSpace. The mean silhouette is usually used to evaluate the clusters. Generally, a silhouette value above 0.5 indicates that the cluster is reasonable. If it is over 0.7, the cluster is believed to be highly effective and persuasive. In this study, we found seven clusters (Table 4, Figure 6). The total silhouette value exceeded 0.7, while that for each cluster was above 0.5, implying a high degree of credibility in the obtained outcomes. This study's modularity (*Q*) is 0.3684 (>0.3), indicating that the clustering structure was considered substantial.

**Table 4:** Keyword cluster analysis.

**Cluster ID**	**Silhouette**	**Label (LLR)**	**Included keywords (top 5)**	**Mean (Year)**
0	0.763	Alzheimers disease	Alzheimers disease; parkinsons disease; restless legs syndrome; sleep disorders; bipolar disorder	2011
1	0.772	Sleep quality	Sleep quality; chronotype; actigraphy; patterns; circadian clock	2010
2	0.768	Bright light	Bright light; melatonin; jet lag; light; light exposure	2008
3	0.643	Circadian clock	Circadian clock; gene expression; clock genes; rhythms; clock gene	2012
4	0.616	Metabolic syndrome	Metabolic syndrome; obesity; blood pressure; hypertension; insulin resistance	2013
5	0.706	Bipolar disorder	Bipolar disorder; major depressive disorder; mood disorders; sleep deprivation; depression	2011
6	0.821	Pineal gland	Pineal gland; hormone; cortisol; sleep disorders; melatonin	2010

### 3.5. Bibliometric analysis of keywords with citation bursts

**Figure 7:**
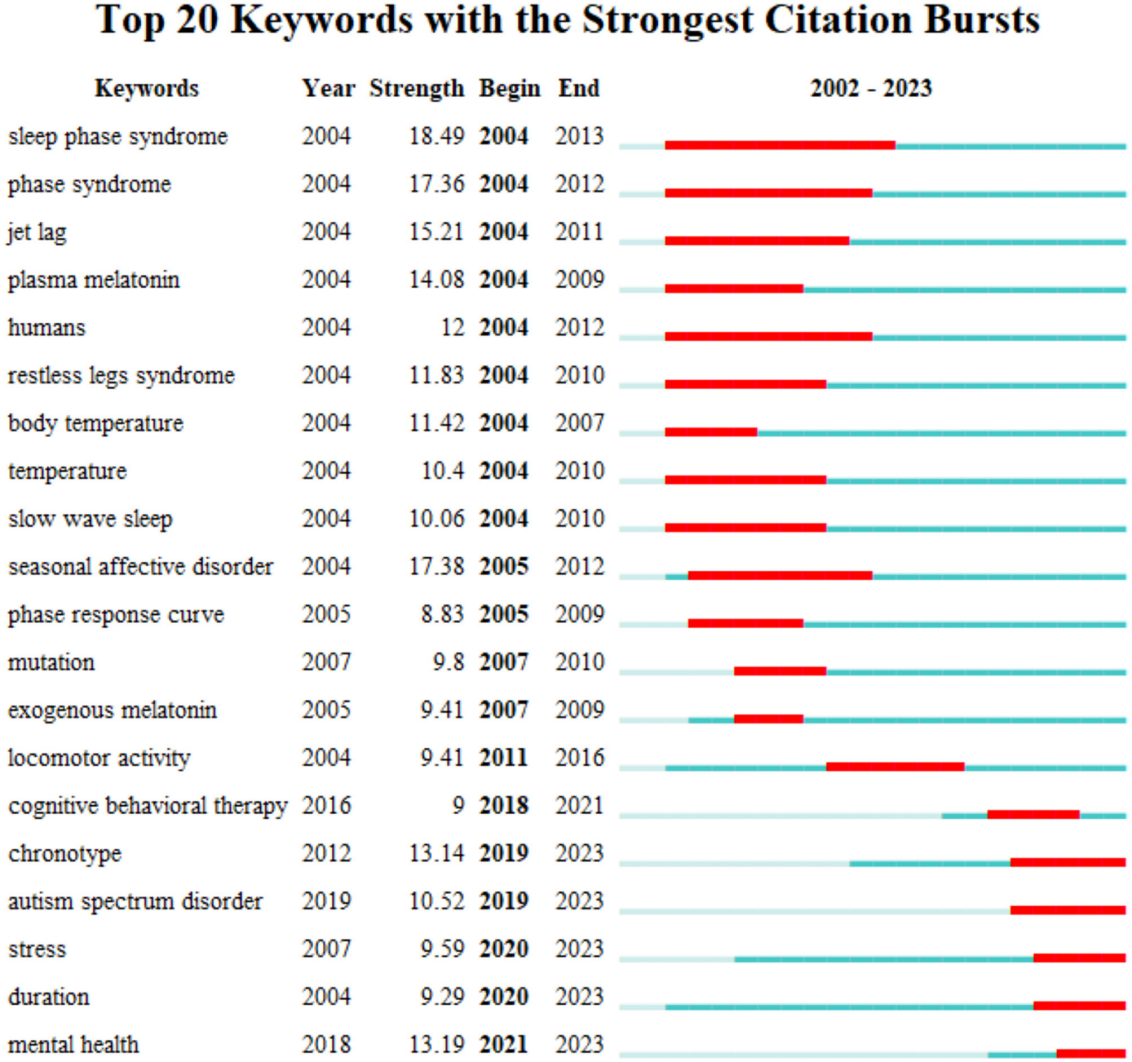
Top 20 keywords with the strongest citation bursts.

The “keywords with citation bursts” denotes keywords that are frequently cited during a specific period. The top 20 keywords with the most potent citation bursts are displayed in Figure 7. The green line represents the whole period and the period during which a keyword's burst was identified is shown by the red line. In 2004, keywords with citation bursts appeared, which were sleep phase syndrome, phase syndrome, jet lag, plasma melatonin, humans, restless legs syndrome, body temperature, temperature, and slow wave sleep. Among them, sleep phase syndrome was the strongest keyword. There were five keywords (chronotype, Autism spectrum disorder, stress, duration, and mental health) with citation bursts till 2023.

### 3.6. Bibliometric analysis of keywords with timeline

**Figure 8:**
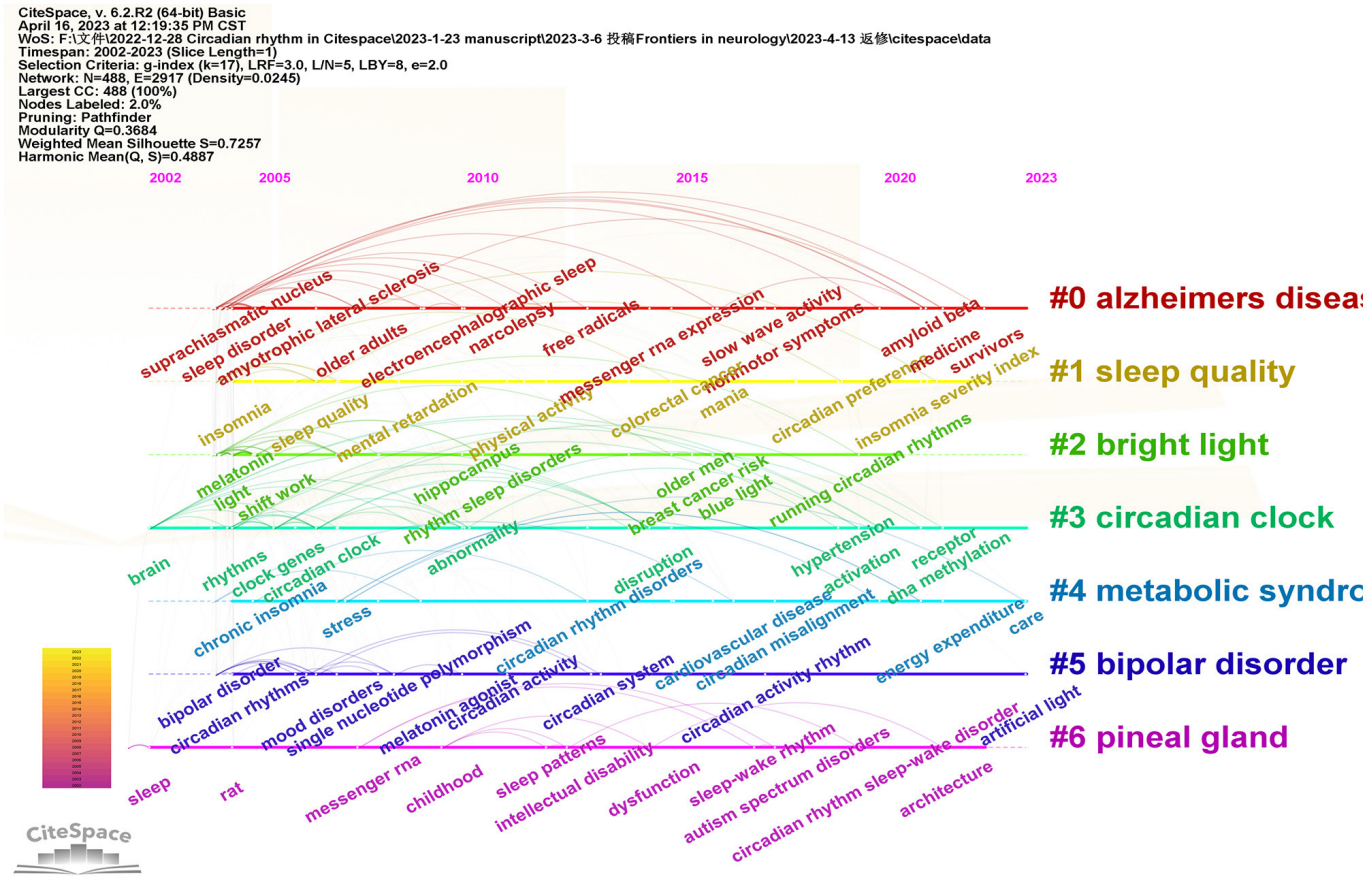
Timeline diagram of keywords in the field of insomnia and circadian rhythm.

Timeline diagrams can reflect the historical evolution and frontiers of hot spots in the research field in recent years. The keyword clustering of research continued up to now mainly involved #0 Alzheimers disease, #1 Sleep quality, #2 Bright light, #3 Circadian clock, #4 Metabolic syndrome, #5 Bipolar disorder, and #6 Pineal gland. As shown in Figure 8, from 2002 to 2005, the keywords in this field focused on brain research, sleep disorders, circadian rhythm, Suprachiasmatic Nucleus, and melatonin, which are the most basic and important research directions. In recent years, keywords such as artificial light and machine learning have appeared. The multidisciplinary intersection is the current hot spot and trend of scientific research, which will significantly promote the development of the depth and breadth of scientific research.

## 4. Discussion

### 4.1. General information

Our study used CiteSpace to review the current state of insomnia and circadian rhythm research, showing its topical and cutting-edge issues. A total of 4,696 articles were retrieved in WoS. The rapid growth rate after 2017 might be closely related to the year when the Nobel Prize in Physiology was awarded to Jeffrey C. Hall, Michael Rosbash, and Michael W. Young for their discovery of the molecular mechanism of the circadian rhythm. After this year, studying life rhythm became one of the hottest spots in medical research. Bruno Etain from Université de Paris had the highest number of articles, 24. The USA and the University of California are leading the research in this field with 1,672 and 269 articles, respectively. Countries and institutions actively cooperated to promote this area of research. The focus of current hot topics is on the interplay between insomnia and circadian rhythms and the range of physical and mental disorders caused.

### 4.2. Hot issues in insomnia and circadian rhythm research

According to the results of co-occurring keywords and cluster analysis, major ongoing research trends include circadian rhythm sleep disorders, the interaction of insomnia and circadian rhythms, and advanced therapies such as melatonin and light therapy.

#### 4.2.1. Circadian rhythm sleep disorders

There are physiological and behavioral circadian variations in mammals. The core biological clock that controls circadian rhythms in humans is located in the suprachiasmatic nucleus (SCN), which generates and maintains circadian rhythms. Genetics governs endogenous circadian rhythms and adapts in response to changes in the natural environment, social factors, and work hours. The most typical of human circadian biorhythms is the sleep–wake cycle. The generation of sleep and wake behaviors depends on the interaction of endogenous circadian rhythms, the process S of internal environmental homeostasis, and social and environmental factors, thus enabling patients to following a pattern of approximately 16 h of the day awake and 8 h of sleep. The daily variation of physiological sleep processes in humans is characterized by a biphasic circadian rhythm toward wakefulness and sleep, with an increase in sleepiness around 2–4 p.m., followed by a significant decrease and an increase in alertness that lasts until midnight. To maintain optimal sleep and alertness, the timing of sleep and wakefulness should be synchronized with the timing of endogenous circadian rhythms; otherwise, circadian rhythm sleep disorders (CRSD) may occur, causing insomnia and/or excessive daytime sleepiness and affecting the quality of life.

The International Classification of Sleep Disorders—Edition 3 (ICSD-3) classifies sleep disorders into seven major categories, including insomnia, sleep-related breathing disorders, central narcolepsy, circadian rhythm sleep–wake disorders, sleep anomalies, sleep-related movement disorders, and other sleep disorders (25). Among them, circadian sleep–wake disorders are caused by the dysregulation of the sleep–wake cycle and the 24-h biological rhythm of the human body. The main clinical manifestations include insomnia, sleepiness, and daytime fatigue; for example, delayed sleep phase disorder (DSWPD), familial advanced sleep phase (FASP), and non-24-h sleep rhythm disorder. Among them, DSWPD is the most frequently diagnosed sleep disorder, mainly affecting adolescents, young adults, and individuals with insomnia. It is characterized by a persistent inability to fall asleep at earlier, more desirable, and socially conventional times while also experiencing extreme difficulty waking up in the morning. Current treatment methods primarily focus on advancing the biological clock and sleep timing through pharmacotherapy, light therapy, and behavioral therapy (26–28). FASP is a sleep disorder associated with hereditary genetic mutations. Individuals with this condition experience a consistently earlier timing of their biological clock relative to the expected norms, resulting in early sleep onset and wake-up times. ASPD is considered to be one of the autosomal dominant genetic disorders (29–31). Besides, sleep disorders are caused by environmental changes that lead to a mismatch between sleep–wake time and an intrinsic circadian rhythm, such as jet lag or shift work. In addition to the classic sleep diary, actigraphy, a non-invasive method of monitoring human rest/activity cycles for activity recording, has become an accepted tool for objective evaluation in the diagnosis of CRSD.

#### 4.2.2. Circadian clock

**Figure 9:**
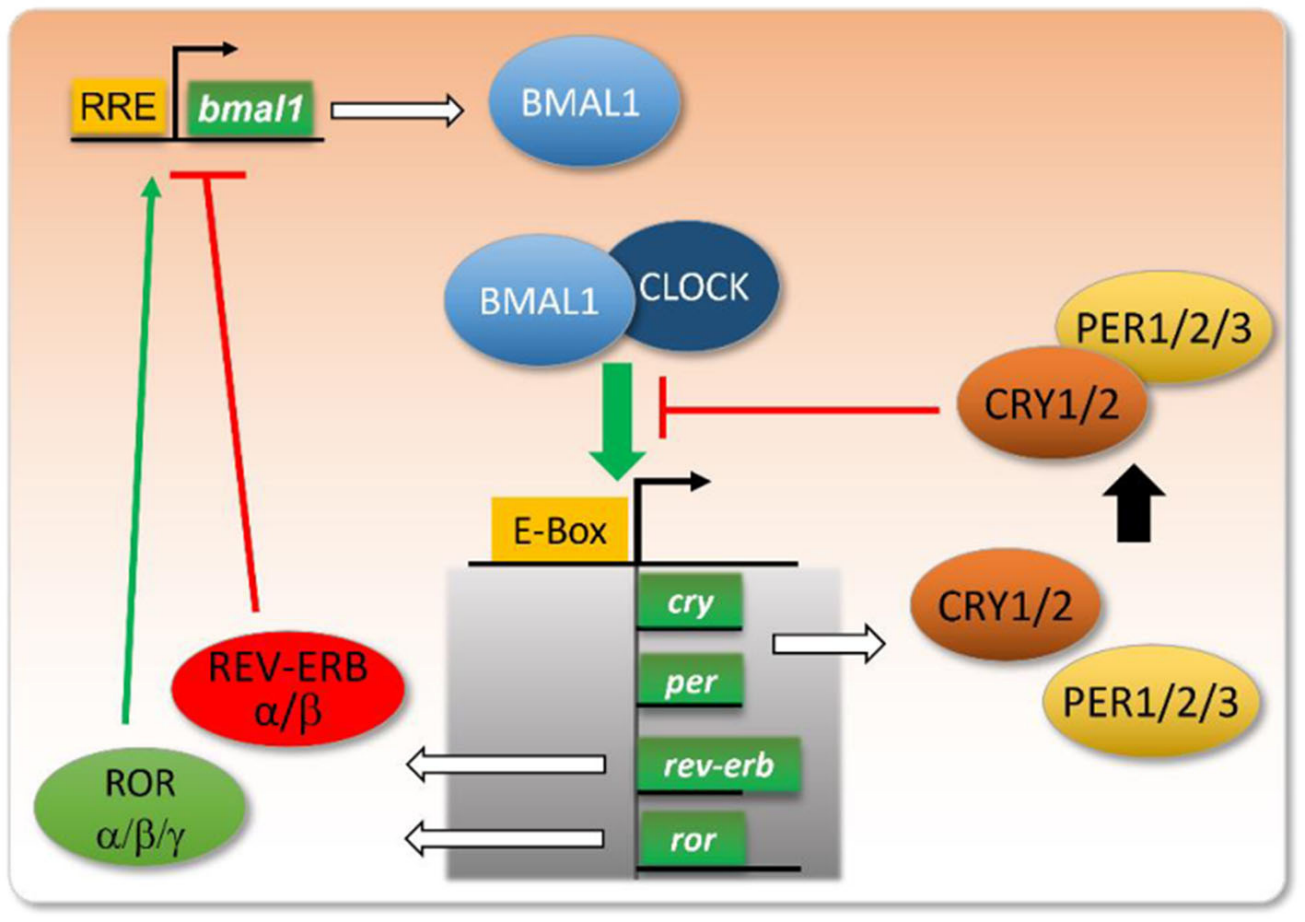
Map of core clock genes and their negative feedback regulation mechanism (32).

The circadian rhythm regulates sleep mainly through clock genes and clock control genes. Clock genes are dominated by Clock genes are dominated by Clock, Bmal1, Per, and Cry (Figure 9). Upon stimulation of the central SCN, which controls circadian rhythm, Clock and Bmal1 first form a CLOCK/BMAL1 heterodimer that binds to the Per gene promoter's E-box element and stimulates transcriptional translation to produce PER protein. When the number of PER1 and PER2 protein monomers exceeds the phosphorylation capacity of cytoplasmic casein kinase 1 (CK1), part of the intact PER protein dimerizes and enters the nucleus, where it binds to the intranuclear protein CRY to form a complex that removes the CLOCK/BMAL1 dimer bound by the E-box element, thereby inhibiting Per gene transcription. Conversely, decreasing PER levels in the nucleus will restart Per gene transcription (32). These processes form a feedback loop through transcriptional and posttranscriptional regulation to generate molecular oscillations that regulate the rhythmic expression of downstream proteins and their physiological functions.

Clock genes exhibit a robust correlation with DSWPD, particularly, *CRY1*. A case report has demonstrated that mutations identified in the exon 11 allele of *CRY1* extend the duration of circadian molecular rhythms, thereby delaying both sleep onset and wakefulness, ultimately leading to the beginning of DSWPD, a phenomenon characterized by individuals who habitually go to bed late and wake up late—commonly referred to as “night owls.” This sleep disorder may also possess a genetic predisposition (33). Furthermore, DSWPD is also influenced by *Per3*. The H4 haplotype of the *Per3* renders the population susceptible to this malady (34). Interestingly, *CRY2* mutation can abbreviate the duration of circadian molecular rhythms, thus triggering FASP, which induces people to have shorter sleep–wake cycles, leading to early morning awakenings and being alert in the evening. These findings imply that clock genes play an integral role in regulating human sleep–wake patterns (35).

The clock control genes consist of Rev-erbα, Rorα, Dbp, Tef, Hlf, etc., which form a regulatory system with close interconnection and interlocking feedback loops (32). Bmal1 and Clock or its homolog NPAS2 form a heterodimer. The Bmal1:Clock/NPAS2 complex binds to Per1-3 and Cry1/2 promoters by binding to the E-box elements on their promoters, thereby activating their transcription. When Per and Cry accumulate to a certain level in the cytoplasm, they dimerize and bind to the Bmal1:Clock/NPAS2 complex in the nucleus, thereby inhibiting their activity and shutting down their transcriptional processes. In addition, this master feedback loop is interconnected with several regulatory loops involving nuclear receptors, such as the orphan nuclear receptor ROR (α, β, γ) (RAR-related orphan receptor α/β/γ) and REV-ERB (α, β) (nuclear receptor subfamily 1 group D member α/β). Bmal1 drives the transcription of Rorα and Rev-erbα and, in turn, Rorα and Rev-erba competitively enhance and repress the transcriptional levels of Bmal1 (36–39). These feedback loops constitute a complex regulatory network that rhythmically regulates the expression of many target genes, such as Bmal1:Clock/NPAS2 heterodimer that regulates the E-box element that regulates the transcription factors Dbp (D-box binding protein), Tef (thyrotrophin embryonic factor), Hlf (hepatic leukemia factor), and Tef (hepatocyte). Hepatic leukemia factor), and Klf10 (Krüppel-like factor 10) (40, 41), which in turn can form homodimers or heterodimers and subsequently activate the expression of downstream target genes (42).

Furthermore, mammals also possess regulatory genes that play an indispensable role in maintaining a regular sleep–wake cycle, such as *Dec1* and *Dec2*. These genes can be found in differentiated embryonic chondrocytes or chondrocytes and suppress Clock/Bmal1-induced transcription of the Per1 promoter. It is worth noting that these regulatory proteins are closely associated with the duration of sleep (43, 44). *Dec2* mutation results in a reduced sleep duration in mice, characterized by a truncated non-REM and REM sleep cycle, alongside a more disrupted pattern of sleep duration (45). Moreover, there exist genes that are linked to sleep homeostasis, such as *ADRB1* (46) and *NPSR1* (47), whose mutation is correlated with individuals having a shorter sleep duration. These three genes may offer an explanation as to why shorter sleepers require less sleep, yet they do not suffer any noticeable impact on their concentration or creativity at work.

#### 4.2.3. Light therapy

Light affects the optic chiasm SCN through the eye and retinal-thalamic tract, delaying circadian rhythms and early morning awakening in patients with insomnia. In addition, light stimulation to the retina suppresses sympathetic nerve activity and inhibits melatonin synthesis (48). In recent years, light therapy has been used clinically as a non-pharmacological treatment for patients with insomnia, especially for circadian rhythm sleep disorders (49–51). During the treatment, subjects were asked to wear blue-light-rich light glasses or direct blue–green light during waking hours (52–54). The study results showed that light therapy alleviated difficulty falling asleep, drowsiness, and daytime fatigue in insomnia patients, a result that was more pronounced in Alzheimer's patients. In addition, the effect of light therapy on circadian rhythms was more significant than the effect on sleep, which may be related to the ability of light to affect the function of the SCN and melatonin secretion.

Light therapy is a natural and simple treatment which does not cost much. Compared to conventional medication, light therapy does not lead to residual effects and tolerance (55, 56). However, light therapy may also have side effects such as headache, eye fatigue, and overactive autonomic nerves, and the therapy may also induce mania (57).

#### 4.2.4. Melatonin

Melatonin is a substance isolated from bovine pineal gland extract by American dermatologist Lerner in 1959 that causes skin discoloration in frogs. After melatonin is synthesized and stored in the pineal gland, sympathetic excitation innervates the pineal cells to release melatonin. Its secretion appears to vary in a distinct circadian rhythm, with decreased secretion during the day and increased secretion at night.

Melatonin has been shown to regulate the biological clock, induce sleep, regulate the endocrine system, maintain the stability of the internal environment, enhance immunity, and delay aging. Among the various regulatory effects of melatonin, its role in sleep regulation is prominent. It has an essential role in treating delayed sleep syndrome, jet lag, and shift sickness (58). Melatonin regulates sleep by interacting with the receptors MT1 and MT2, which are members of the G protein-related receptor family, mainly found in the SCN. The two receptors act differently, with MT1 inhibiting neuronal activity and MT2 mainly inducing phase shifts (59). Exogenous melatonin supplementation is effective for insomnia caused by reduced melatonin secretion, and oral melatonin can improve sleep quality in the elderly with a better safety profile. Therefore, melatonin is often used to treat circadian sleep disorders, including delayed sleep phase syndrome, jet lag syndrome, shift workers, sleep–wake rhythm disorder, and non-24-h sleep–wake syndrome.

Melatonin has a short half-life of approximately 40 min and is metabolized rapidly in the body. Recently, the European Medicines Agency has approved the use of 2 mg of extended-release melatonin as a short-term treatment for patients over 55 years of age with primary insomnia. Its efficacy is limited for most sleep disorders due to its short half-life and poor absorption in the body when taken orally. In recent years, melatonergic drugs have been developed to treat insomnia more effectively, and the most studied drugs are ramelteon and agomelatine. They have become a hot research topic in the treatment of sleep disorders.

#### 4.2.5. Bipolar disorder

Bipolar disorder is a chronic relapsing disorder whose clinical manifestations are characterized by intermittent episodes of depression, mania, and hypomania (60). The disorder affects more than 1% of the world's population and is one of the main reasons why young people have cognitive deficiencies, functional impairment, and disability and eventually die (61, 62). Circadian rhythm disturbances are one of the clinical manifestations of bipolar disorder, including sleep–wake rhythms, activity rhythms, body temperature rhythms, blood pressure, pulse, urine output, norepinephrine, and hormone secretion (63). Patients with bipolar disorder show reduced sleep demand during mania, mainly insomnia or drowsiness during depressive episodes. Sleep disturbance can also occur even in the remission period of bipolar disorder (64). The pathogenesis of bipolar disorder is still unclear. Some studies have found that bipolar disorder is correlated with clock genes, such as the Per gene, Clock gene, and Cry gene mentioned before (65–68). Therefore, the regulation of circadian rhythms is an essential part of the treatment of bipolar disorder, and studies have found that Interpersonal and Social Rhythm Therapy (IPSRT) (69, 70), light therapy (71), and lithium therapy (72) can improve the clinical symptoms of bipolar disorder by regulating the circadian rhythms of patients.

### 4.3. Strengths and limitations

To the best of our knowledge, this is the first study that used CiteSpace to undertake bibliometric analysis, visual display of insomnia and circadian rhythm from hot spots and frontiers, and collaboration among authors, countries, and institutions. However, there still exist some limitations. We only analyzed English studies in WoS. Because of this, the data may be less comprehensive. In addition, because there were several synonyms, there might be some overlap between various content categories when clustering keywords.

## 5. Conclusion

Based on the CiteSpace results, we recommend a more active collaboration between various countries, institutions, and authors to conduct clinical and basic research. Ongoing research is focused on the interaction of insomnia with circadian rhythms and the corresponding pathways of clock genes and, consequently, the role of circadian rhythms in disorders such as bipolar disorder. Modulating circadian rhythms may be a hot spot for future insomnia therapies (such as light therapy and melatonin).

## Data availability statement

The original contributions presented in the study are included in the article/supplementary material, further inquiries can be directed to the corresponding authors.

## Author contributions

Q-YH designed and analyzed the data. Q-YH and ND drafted the manuscript and edited the manuscript. Q-YH and QW analyzed the data. ND, MM, JM, YL, and FL contributed to revising the manuscript. All authors contributed to the article and approved the submitted version.
